# How Can the Clinical Aptitude of AI Assistants Be Assayed?

**DOI:** 10.2196/51603

**Published:** 2023-12-05

**Authors:** Arun James Thirunavukarasu

**Affiliations:** 1 Oxford University Clinical Academic Graduate School University of Oxford Oxford United Kingdom; 2 School of Clinical Medicine University of Cambridge Cambridge United Kingdom

**Keywords:** artificial intelligence, AI, validation, clinical decision aid, artificial general intelligence, foundation models, large language models, LLM, language model, ChatGPT, chatbot, chatbots, conversational agent, conversational agents, pitfall, pitfalls, pain point, pain points, implementation, barrier, barriers, challenge, challenges

## Abstract

Large language models (LLMs) are exhibiting remarkable performance in clinical contexts, with exemplar results ranging from expert-level attainment in medical examination questions to superior accuracy and relevance when responding to patient queries compared to real doctors replying to queries on social media. The deployment of LLMs in conventional health care settings is yet to be reported, and there remains an open question as to what evidence should be required before such deployment is warranted. Early validation studies use unvalidated surrogate variables to represent clinical aptitude, and it may be necessary to conduct prospective randomized controlled trials to justify the use of an LLM for clinical advice or assistance, as potential pitfalls and pain points cannot be exhaustively predicted. This viewpoint states that as LLMs continue to revolutionize the field, there is an opportunity to improve the rigor of artificial intelligence (AI) research to reward innovation, conferring real benefits to real patients.

## Introduction

The development of large language models (LLMs) with remarkable performance in unseen tasks introduces the possibility of artificial intelligence (AI) assistants participating in health care delivery—applications with general knowledge and skills in contrast to existing AI applications with narrow use cases [1,2]. LLMs such as GPT-4 (Generative Pretrained Transformer 4; OpenAI) and Med-PaLM (Pathways Language Model) 2 (Google) are pretrained on billions of human-generated words in context before being fine-tuned to optimize responses to user-generated queries [3]. LLMs have attained expert-level performance in United States Medical Licensing Examination sample questions, and while reported performance in tests for fully qualified specialists has not been as high, progress seems inevitable [3-6]. When pitted against qualified doctors working in their spare time to answer patients’ queries on a social media platform, ChatGPT provided more accurate and empathetic responses according to a blinded panel of clinically trained judges [7]. However, the above experiments may not evidence actual clinical aptitude. This viewpoint aims to stimulate greater effort toward clinical research—particularly, randomized controlled trials (RCTs)—to validate clinically useful tools to improve patient outcomes.

## Can a Benchmark Provide Evidence of Clinical Aptitude?

LLM experiments have generally used unvalidated surrogate variables to appraise clinical aptitude [3]. In particular, examination performance has been widely reported and has attracted major attention, especially as major developers use it as a benchmark indicator of clinical knowledge and skill [4,6,8]. This may reflect the ease of investigation and comprehension of examination results. However, these results may not translate to clinical performance—examinations are used alongside a myriad of other forms of assessment of doctors, including written and practical tests as well as mandatory experience from medical school to completion of specialty training and beyond. While impressive examination performance may indicate clinical potential, it does not constitute evidence that an LLM (or a person) can practice safely. Even as examination performance approaches the level of experts, fact fabrication and inaccuracies remain as contraindications to autonomous deployment [1]. These issues may be ameliorated through system design, such as oversight by physicians, but this requires validation demonstrating true benefit (or at the very least, nonharm) to patients and practitioners [1,3]. Could another benchmark be designed to explore clinical potential before high-risk tools are deployed in health care?

Ayers et al [7] leveraged a social media message board to conduct a comparative study of LLM and doctor responses to patient queries, finding that AI-generated answers were generally preferable in terms of response “quality” and “empathy,” as graded by a panel of blinded doctors. Similarly, Google used 9 qualitative variables to compare the performance of Med-PaLM 2 with physicians in responding to a data set of questions patients could ask, finding the LLM to be superior overall [6]. The artificial or unofficial settings of these studies hinder the extrapolation of these results to any specific clinical setting. Moreover, risks such as bias, fact fabrication, and inconsistent output preclude immediate clinical deployment [3,9]. However, these studies retain the strengths of examination-based experiments by exposing human and LLM to identical tasks for fair comparison of performance while improving on previous studies by bringing AI into a more realistic setting [10-12].

## The Argument for Randomization

Without RCTs, the effect of LLM deployment in clinical settings is unknown. An LLM *may* generate better responses than clinicians on average, and accuracy *may* be excellent when compared to expert opinion, guidelines, and relevant research [7]. LLMs *may* imitate a good clinician perfectly. However, it does not logically follow that such systems are safe or beneficial to patients if implemented as an autonomous chatbot to dispense advice to patients or as a source of expertise for clinicians to draw on in uncertain conditions. There are too many intangible and unknowable variables at play. Do the models have pain points leading to error, and what is the relative impact of these on patient outcomes at scale? What are the effects on patients on implementing yet another barrier to accessing care from a human clinician? How are practitioners and the care they deliver affected by referring to a computational model rather than a trusted colleague for advice? All of these questions and more could feasibly have adverse effects on outcomes following implementation.

The old ways are the best: no benchmark can replace RCTs. Through randomization, we may reliably determine the effect magnitude and (more importantly) direction conferred by implementing autonomous AI. The effect of confounding variables is equilibrated between experimental groups, and all of the known and unknown effects of the AI intervention are trialed. Differences in outcome between the groups—positive or negative—may thereby be confidently attributed to the intervention, mirroring the benefit of a consistent benchmark that underlies the methodology of existing experiments using LLM applications. Mortality and morbidity outcomes can minimize bias and facilitate power analysis based on the estimated disease burden of medical error and inaccessibility of care—the issues that the AI intervention is designed to solve [13,14]. In terms of design, preferences may differ: cluster RCTs would reduce the risk of crossover, but randomizing patients or practitioners within centers would compensate for performance bias that could affect results.

Despite the proliferation of medical AI research generating remarkable hype around the subject, very few clinical trials have shown AI to improve outcomes when compared with standard clinical practice [15]. Most studies are retrospective and exhibit highly curated settings, resulting in a high risk of bias [15]. In general, there is too generous a reward in terms of publications, citations, and funding for discussion and demonstration of *potential* clinical applications rather than what really matters for patients and practitioners: tangible improvement of clinical outcomes and workflows. As LLM applications emerge, we have an opportunity to break this trend, shifting medical AI research toward truly impactful aims and methods.

## Conclusions: Designing the Future

It is now conceivable that AI models with superior clinical reasoning and communication aptitude to most clinicians can be developed, and it follows that these models should influence or even determine courses of action taken in medicine and surgery [1,7]. The implementation of clinical AI assistants would be a pivotal moment in health care, and it is critical that patients are safeguarded accordingly. Not all RCTs are created equal, with bias, fraud, and opaque reporting all reducing confidence in conclusions [16]. However, well-designed pragmatic RCTs evidencing the benefit of an intervention are the only acceptable justification for the deployment of such influential applications, provided ethical concerns surrounding implementation are addressed.

This stringent requirement will attenuate the recent hype around medical AI. Greater cognitive work is required to design a pragmatic trial than merely inputting existing material (repurposed as a benchmark) to an LLM. The intervention must be conceptualized and designed with specific regard for how LLMs may be implemented in clinical workflows. Development should therefore focus on improving specific aspects of health care rather than attaining higher performance on an arbitrary benchmark. Successful applications will likely restructure rather than replace care provided by humans [1]. Patients and practitioners must demand the highest standard of evidence to ensure that innovative developments represent more than mere hype.

## Abbreviations

AI: artificial intelligence
GPT-4: Generative Pretrained Transformer 4
LLM: large language model
PaLM: Pathways Language Model
RCT: randomized controlled trial
